# Prolapse of ectopic ureterocele in the vulva

**DOI:** 10.1016/j.eucr.2023.102425

**Published:** 2023-05-10

**Authors:** Boumediene Abou-Bekr, Yamina Ouadah, Soumia Chikh Bled, Chahrazed Boghari, Hayat Meftah

**Affiliations:** a Department of Pediatric Surgery, Mother and Child Specialty Center, Universite Abou Bekr Belkaid Tlemcen Faculte de Medecine, Algeria; b Department of Paediatrics, Mother and Child Specialty Center, Universite Abou Bekr Belkaid Tlemcen Faculte de Medecine, Algeria; c Department of Resuscitation, Universite Abou Bekr Belkaid Tlemcen Faculte de Medecine, Algeria

**Keywords:** Ureterocele, Ureteral duplicity, Vulvar swelling

## Abstract

Ureterocele is rare urinary malformation. We reported a 12-month-old girl case with a prolapsed ectopic ureterocele in the vulva. Urinary catheterization was used as an emergency treatment to minimize the ureterocele. The patient later benefited from an upper polar hemi-nephrectomy, which helped to resolve the issue partially. She sought treatment for the prolapse again a Three month later, this time ureterecelectomy with ureteral reimplantation was employed. The non-consensual management of this malformation must be initiated as soon as possible to prevent complications. The first treatment goal was to decompress the prolapsed ureterocele and remove it endoscopically.

## 1 Introduction

Ureterocele is a rare congenital urinary malformation. Enriching the iconography employed for ectopic ureterocele detection is crucial for early or even antenatal diagnosis, in order to adopt adequate care before the occurrence of complications. We report a case of a prolapsed swelling of the vulva to describe a North African infant presenting with a prolapsed ureterocele without prior prenatal diagnosis and highlight the need for providing prenatal care in low- and middle-income countries.

## 2 Case presentation

**Fig. 1:**
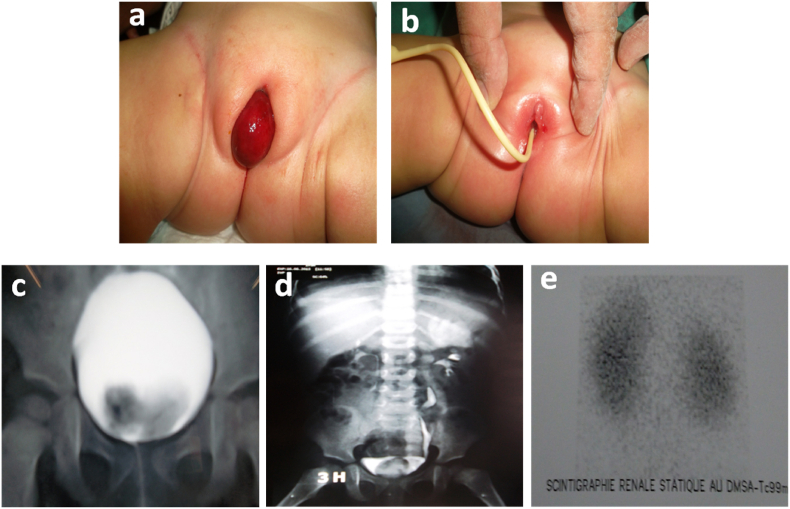
(a) Prolapsed mass in the vulva. (b) Reduction of prolapsed and urinary catheterization. (c) Intravesical lacunar image at the (VCUG). (d) Left ureteral duplicity on (IVU). (e) Upper left polar hypofixation.

**Fig. 2:**
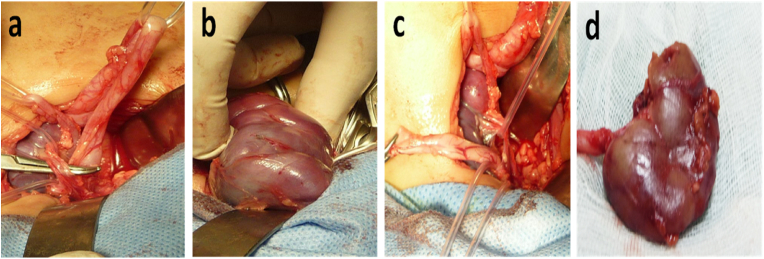
Left upper polar heminephrectomy. (a) Identification of the ureters and the vessels of the upper lobe on lake. (b) Clamping test of the superior polar artery to delimit the area to be resected. (c) Aspiration and drying of the ureterocele. (d) Upper polar heminephrectomy.

**Fig. 3:**
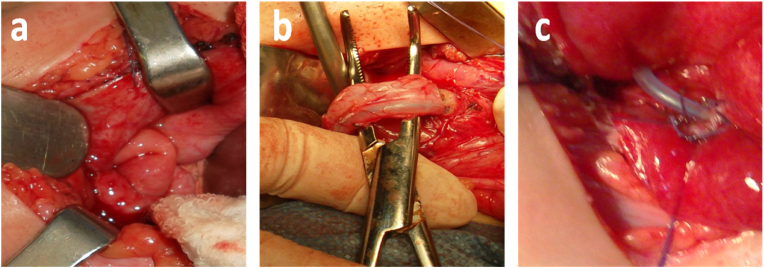
Ureterocelectomy and reimplantation of 02 ureters. (a) Individualization of the ureterocele. (b) Dissection of the ureters. (c) Reimplantation of the 02 gun barrel ureters.

A 12-month-old girl consulted in the emergency room for a red, smooth, inflammatory vulvar swelling prolapsed through the urethral meatus (Fig. 1a). Gentle manual reduction performed in an emergency with bladder sounding allowed reintegration of the prolapse (Fig. 1b). A series of radiological examinations (ultrasound of the urinary tract, voiding cystourethrogram (VCUG) (Fig. 1c) and intravenous urography (IVU) (Fig. 1d), revealed an ectopic ureterocele on a left pyelo-ureteral duplicity with a dumb superior renal pole (non-functional) in renal scintigraphy (Fig. 1e). The patient underwent a left upper polar hemi nephrectomy with aspiration and drying of the ureterocele (partial treatment) (Fig. 2). A month later, the prolapse reappeared, causing recourse to radical treatment by ureterocelectomy with ipsilateral ureterovesical reimplantation in gun barrel (radical treatment) (Fig. 3). The immediate and late postoperative evolutions were favorable and satisfactory. The monitoring of the girl was clinical and biological noted a disappearance of the urinary tract infection with cytobacteriological studies of negative urine as well as abdominal ultrasounds carried out every 6 months which were normal.

## 3 Discussion

Ureterocele is a cystic dilatation of the distal ureter that can have a variety of morphology and symptoms. The estimated prevalence of ureteroceles in children is highly variable due to the existence of asymptomatic forms. Prenatal ultrasound is being used more frequently to diagnose these ureteroceles in utero; their prevalence at this stage is estimated to be 1 in 5000–9000 live births. In 10% of cases, they are bilateral and 4 to 7 times more common in girls, with a little left-side predilection.

Orthotopic ureterocele, which is totally inside the bladder, is distinguished from ectopic ureterocele, which may be in the bladder but has a portion that is always below or at the level of the bladder neck. The results of radiographic and cystoscopic exams form the basis for this classification. Additionally, the clinical expression might range from asymptomatic forms to problems.^1^^,^^2^ Differentiating prolapsed ureterocele from other vulvovaginal swellings is crucial. Nowadays, most ureteroceles are diagnosed in utero by the discovery of uretero-hydronephrosis on obstetric ultrasound by looking for ipsilateral pyelo-ureteral duplicity. However, definitive prenatal diagnosis relies on the identification of distal ureter dilatation protruding into the bladder, giving the characteristic balloon-like appearance in the bladder. Nevertheless, fetal ureteroceles can easily escape ultrasound detection.

Prenatal diagnosis significantly reduced the morbidity of ureteroceles and the frequency of urinary tract infections, only 12–20% were reported when the ureterocele was diagnosed in utero. Apart from prenatal diagnosis, 90% of ureteroceles are diagnosed before the age of 3 years, during investigations of a urinary tract infection which remains the most frequent discovery circumstance.

Definitive diagnosis and preoperative evaluation of ureteroceles are based on the urinary tract radiological and isotopic explorations. Although ultrasound is a non-invasive imaging tool, it has limits and risks ignoring discrete dilatations, ureteral unicity or duplicity as well as kidney functional value. While intravenous urography can confirm ureteral unicity or duplicity, currently supplanted by magnetic resonance urography, which is rapid and contributes significantly to the ureteroceles diagnosis by allowing the renal function evaluation and providing higher resolution images, especially when urinary tract dilation is low.^3^ Once the diagnosis of ureterocele is confirmed, retrograde urethrocystography must be systematic in search of vesicoureteral reflux and assess its degree.

Retrograde urethrocystography is still useful for detecting any new reflux that may develop after an ureterocele has been repaired endoscopically in more than 50% of afflicted individuals, suggesting that the contralateral system may also be affected in roughly 25% of these patients.

A DTPA kidney scan is always needed to accurately assess kidney function and plan definitive surgery. Kidney scans with dimercaptosuccinic acid (DMSA) or mercaptoacetyltriglycine (MAG3) are used to evaluate the distribution function in the duplex kidney.

Finally, cystoscopy remains a considerable contribution, highlighting the ureterocele in the form of a contractile pseudocystic formation and contributing to the choice of endoscopic or open surgical treatment. Recent progress has greatly improved the postnatal management of ureteroceles. The individualization of the ureteroceles treatment is always useful for practical management, with no single surgical method suitable for this anomaly and the challenges in this regard persist. Among the many available therapeutic means, the endoscopic puncture remains the least invasive and the most recommended in first intention. Additionally, laser ablation is a safe and effective therapy for the ureteroceles management in the neonatal period and remains an important alternative to electrosurgery. The revision rate after orthotopic ureteroceles elective endoscopic puncture ranges from 3.8 to 4.7%.^4^ As the upper pole renal function that drains the ureterocele is either absent or severely impaired in over 90% of cases, definitive treatment usually involves upper pole heminephrectomy and subtotal ureterectomy with excision of ectopic ureteroceles ranges from 15 to 73.7%.^5^

In case of ectopic ureterocele with vesicoureteral reflux, ureterocele resection with ureter reimplantation must be performed immediately, regardless of initial treatment.^5^ In the literature, the average age at the initial intervention in the case of ureteroceles on simplex ureters varies from 26 to 34 months^4^, whereas in the case of ureteroceles on the double system, the intervention is performed at an average age of 4–9 months. Although endoscopic puncture of the ureteroceles is a good therapeutic alternative, patients must be followed for a long time.

## 4 Conclusion

On a dual system, prolapsed ureterocele at the urethral meatus is a rare entity whose emergency treatment is based on decompression followed by a permanent surgical cure. Although there is no consensus on this matter, the endoscopic route remains the first-line treatment when starting early before any impact on the kidney.

## Declaration of competing interest

The authors declare that they have no known competing financial interests or personal relationships that could have appeared to influence the work reported in this paper.
